# Classifying the antibody-negative NMO syndromes: Clinical, imaging, and metabolomic modeling

**DOI:** 10.1212/NXI.0000000000000626

**Published:** 2019-10-28

**Authors:** Tianrong Yeo, Fay Probert, Maciej Jurynczyk, Megan Sealey, Ana Cavey, Timothy D.W. Claridge, Mark Woodhall, Patrick Waters, Maria Isabel Leite, Daniel C. Anthony, Jacqueline Palace

**Affiliations:** From the Department of Pharmacology (T.Y, F.P., M.S., D.C.A.), University of Oxford, UK; Department of Neurology (T.Y.), National Neuroscience Institute, Singapore; Nuffield Department of Clinical Neurosciences (M.J., A.C., M.W., P.W., M.I.L., J.P.), John Radcliffe Hospital, University of Oxford, UK; Department of Chemistry, (T.D.W.C.), Chemistry Research Laboratory, University of Oxford, UK.

## Abstract

**Objective:**

To determine whether unsupervised principal component analysis (PCA) of comprehensive clinico-radiologic data can identify phenotypic subgroups within antibody-negative patients with overlapping features of multiple sclerosis (MS) and neuromyelitis optica spectrum disorders (NMOSDs), and to validate the phenotypic classifications using high-resolution nuclear magnetic resonance (NMR) plasma metabolomics with inference to underlying pathologies.

**Methods:**

Forty-one antibody-negative patients were recruited from the Oxford NMO Service. Thirty-six clinico-radiologic parameters, focusing on features known to distinguish NMOSD and MS, were collected to build an unbiased PCA model identifying phenotypic subgroups within antibody-negative patients. Metabolomics data from patients with relapsing-remitting MS (RRMS) (n = 34) and antibody-positive NMOSD (Ab-NMOSD) (aquaporin-4 antibody n = 54, myelin oligodendrocyte glycoprotein antibody n = 20) were used to identify discriminatory plasma metabolites separating RRMS and Ab-NMOSD.

**Results:**

PCA of the 36 clinico-radiologic parameters revealed 3 phenotypic subgroups within antibody-negative patients: an MS-like subgroup, an NMOSD-like subgroup, and a low brain lesion subgroup. Supervised multivariate analysis of metabolomics data from patients with RRMS and Ab-NMOSD identified myoinositol and formate as the most discriminatory metabolites (both higher in RRMS). Within antibody-negative patients, myoinositol and formate were significantly higher in the MS-like vs NMOSD-like subgroup; myoinositol (mean [SD], 0.0023 [0.0002] vs 0.0019 [0.0003] arbitrary units [AU]; *p* = 0.041); formate (0.0027 [0.0006] vs 0.0019 [0.0006] AU; *p* = 0.010) (AU).

**Conclusions:**

PCA identifies 3 phenotypic subgroups within antibody-negative patients and that the metabolite discriminators of RRMS and Ab-NMOSD suggest that these groupings have some pathogenic meaning. Thus, the identified clinico-radiologic discriminators may provide useful diagnostic clues when seeing antibody-negative patients in the clinic.


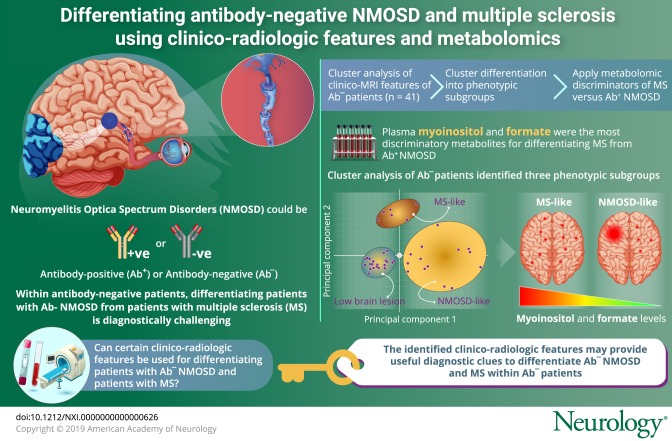


In the multiple sclerosis (MS) or neuromyelitis optica spectrum disorders (NMOSD) clinic, one of the greatest diagnostic challenges is differentiating antibody-negative patients with NMOSD from those with opticospinal MS. This conundrum was demonstrated when large diagnostic disagreement was shown even among experts in this field, despite having the 2015 NMOSD diagnostic criteria; in fact, the criteria were not consistently used.^1^

It is clear that the use of discriminatory models on plasma metabolites or conventional MRI can distinguish patients with relapsing-remitting MS (RRMS) from those with aquaporin-4 antibody (AQP4-Ab) NMOSD and RRMS from myelin oligodendrocyte glycoprotein antibody (MOG-Ab) disease remarkably accurately.^2–4^ Thus, we aim to use these methods to tackle the diagnostic difficulties in antibody-negative patients who have features overlapping NMOSD and MS. The primary methodologic barrier to identifying discriminators of MS and primary antibody-mediated NMOSD is the lack of a gold standard diagnostic tool to test accuracy against. Therefore, there is no published study to date to resolve this clinical dilemma. Given that the treatment of MS and antibody-mediated NMOSD is markedly different, and many MS-specific therapies can worsen antibody-mediated NMOSD,^5–12^ it is paramount that neurologists are able to identify individuals who have antibody-mediated pathology and those with MS pathology, within antibody-negative patients presenting with overlapping clinico-MRI features.

In this study, we aim to classify a group of difficult-to-diagnose, antibody-negative patients into those whose underlying pathology are antibody-mediated and those who are likely to have MS. First, we assess whether there are spontaneous clusters of these patients based on their clinical and MRI features using principal component analysis (PCA). Next, we explore whether these clusters appear to segregate into plausible disease-specific groups. If these spontaneous clusters appear to identify “MS-like” and “NMOSD-like” cohorts, we then apply the metabolomics discriminators of MS vs antibody-positive NMOSD (Ab-NMOSD) (obtained by combining AQP4-Ab and MOG-Ab patients) to further validate that these spontaneous clusters are likely to be representing underlying pathologic processes. If the metabolic differentiators do support the spontaneous clinico-radiologic clusters, one could use the most important differentiating clinico-MRI features when making diagnostic and treatment decisions on antibody-negative patients in the clinic.

## Methods

### Study participants and clinico-radiologic data

**Figure 1:**
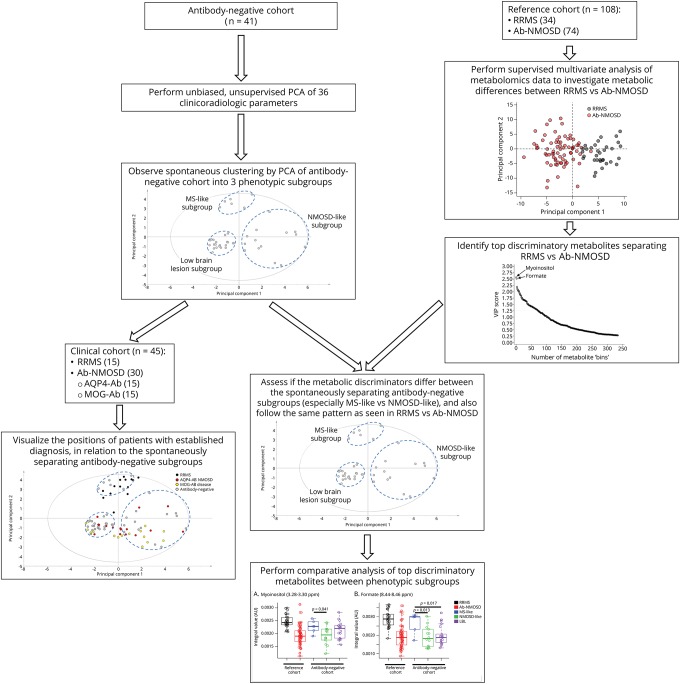
Outline of the study workflow Ab-NMOSD = antibody-positive NMOSD; AQP4-Ab = aquaporin-4 antibody; AU = arbitrary units; LBL = low brain lesion; MOG-Ab = myelin oligodendrocyte glycoprotein antibody; NMOSD = neuromyelitis optica spectrum disorder; PCA = principal component analysis; RRMS = relapsing-remitting MS; VIP = variable importance in projection.

The study workflow is outlined in figure 1.

#### Antibody-negative cohort for PCA model building using clinico-MRI features

Forty-one antibody-negative patients were recruited from the Oxford national NMO service at the John Radcliffe Hospital from November 2013 to September 2015. All patients were out of relapses and were referred by their primary neurologists for possible NMOSD, and none had typical MS. Serum in all patients was negative on multiple occasions for both AQP4-Ab and MOG-Ab, tested by cell-based assays as previously described.^13,14^

Clinico-radiologic data were obtained from medical notes and review of clinical MRIs supplemented by neuroradiologic reports. Thirty-six predefined clinico-radiologic parameters were collected, focusing on features that have been described to distinguish between MS and NMOSD (table e-1, links.lww.com/NXI/A155).^3,4,15,16^ These parameters were scored as present if a patient ever had that clinico-MRI feature. This clinico-radiologic data set was used for unsupervised multivariate PCA for unbiased pattern recognition to identify phenotypic subgroups within the antibody-negative patients (see Statistical analyses).

#### Clinical cohort of patients with RRMS and Ab-NMOSD for visualization of known diagnostic clusters within the PCA model

The same 36 clinico-MRI parameters were collected from 45 patients with established diagnosis (RRMS n = 15, AQP4-Ab n = 15, MOG-Ab n = 15), randomly selected from the Oxford MS/NMO research database. These data were used as a predictive set and inserted into the PCA model that was built using the clinico-MRI data from antibody-negative patients, allowing corroboration of phenotypic subgroups (if any) with known diagnostic clusters.

#### Reference cohort of patients with RRMS and Ab-NMOSD for plasma metabolomics discriminatory analysis

Plasma metabolomics spectral data from an independent cohort of 108 patients with established diagnosis (RRMS n = 34, AQP4-Ab n = 54, MOG-Ab n = 20) was used to build discriminatory models to identify metabolites separating RRMS from Ab-NMOSD (i.e., AQP4-Ab combined with MOG-Ab patients) (see Statistical analyses).^2^ Sample collection protocols were identical, and NMR metabolomics experiments were performed at the same time for both the reference cohort and antibody-negative cohort.

### Standard protocol approvals, registrations, and patient consents

This study was approved by the Oxford Research Ethics Committee C (Ref: 10/H0606/56 and 16/SC/0224A). All patients gave their written consent to participate in the study.

### Plasma collection and NMR sample preparation for metabolomics analysis

Blood was collected into lithium-heparin tubes (Becton Dickinson 367375) and left to stand at room temperature for 30 minutes before centrifugation at 2,200*g* for 10 minutes. Plasma was immediately aliquoted and stored at −80°C. For NMR experiments, plasma was thawed at room temperature, followed by centrifugation at 100,000*g* for 30 minutes at 4°C. One hundred fifty microliters of the plasma supernatant was then diluted with 450 μL of 75 mM sodium phosphate buffer prepared in D_2_O (pH 7.4), followed by centrifugation at 16,000*g* for 30 minutes before transferring to a 5-mm NMR tube.

### NMR spectroscopy and data processing for metabolomics analysis

All NMR experiments were performed using a 700-MHz Bruker AVIII spectrometer. Technical specifications of the NMR experiments and data processing have been previously published.^2^ Briefly, 1D ^1^H NMR spectra were obtained using a Carr-Purcell-Meiboom-Gill (CPMG) relaxation editing pulse sequence, which retains resonances from small-molecular-weight metabolites and mobile side chains of lipoproteins. The CPMG spectra were preprocessed in Topspin 2.1 (Bruker, Germany), followed by visual inspection for errors in baseline correction, referencing, spectral distortion, or contamination. Processed spectra were exported to ACD/Labs Spectrus Processor Academic Edition 12.01 (Advanced Chemistry Development, Inc., Toronto, Canada), whereby regions of the spectra between 0.80–4.20 parts per million (ppm) and 5.20–8.50 ppm were split into 0.02-ppm-wide bins. Integral values of the spectral bins were computed and used as quantitative variables expressed in arbitrary units (AU). Metabolite assignment was performed by referencing to literature values and the Human Metabolome Database.^17–21^ Further confirmation was achieved by inspection of the 2D spectra (presaturation correlation spectroscopy), spiking of known compounds, and 1D total correlation spectroscopy spectra.

### Statistical analyses

To identify potential subgroups within the antibody-negative cohort using clinico-imaging data, PCA was used. SIMCA software (MKS Data Analytics Solutions, Umetrics, Sweden) was used for PCA. PCA is an unsupervised, unbiased (i.e., without defining disease groups) multivariate analysis approach to identify a set of variables (in this case, clinico-MRI parameters) accounting for the greatest variation present in the data set.^22^ As the analysis is unsupervised, clustering (if any) is in no way influenced by the user but rather is wholly dependent on the clinico-MRI data alone. Furthermore, the PCA approach allows the inclusion of correlated variables, which reflects the actual, real-life clinico-MRI (often correlated) data gathered by a neurologist when seeing a patient. This approach was used to analyze the 36 predefined clinico-radiologic parameters (binary data) to evaluate the degree of clustering between the 41 antibody-negative patients based on clinico-MRI features, enabling clusters (if any) to be identified. Loading plots were generated to visualize the clinico-radiologic parameters responsible for clustering.

To identify metabolic differences between RRMS and Ab-NMOSD using metabolomics spectral data, orthogonal partial least square discriminant analysis (OPLS-DA) statistical methods were used.^2^ R software (R foundation for statistical computing, Vienna, Austria) was used for OPLS-DA, using in-house R scripts and the *ropls* package.^23^ OPLS-DA is an extension of PCA allowing supervised multivariate analysis to explore variables (in this case, metabolites) accounting for class discrimination between user-defined classes.^22^ This approach was used to investigate metabolic differences of patients with RRMS vs Ab-NMOSD (i.e., AQP4-Ab combined with MOG-Ab) from the reference cohort and to identify the key metabolites driving the separation between them. In brief, after correction for unequal class sizes, the metabolomics data were split into a training set (90% of data) and a test set (10% of data). The training set was used to build the model on which the test set was applied to, to determine the predictive accuracy of the model. Ten-fold cross-validation with 100 iterations was performed, creating an ensemble of 1,000 model accuracies. To validate the metabolic separation between the disease groups, the mean accuracy of the ensemble of model accuracies was compared with the mean accuracy of a separate ensemble created by random class assignments.

Analysis of other clinicoimaging and metabolomics data was performed with STATA software (Release 14; StataCorp LP, College Station, TX) and R software. Chi-square tests or Fisher exact tests were used for categorical variables as appropriate, whereas 2-sample *t* test/one-way analysis of variance (ANOVA) with Tukey Honestly Significant Difference (HSD) post hoc correction or Mann-Whitney *U*/Kruskal-Wallis tests were used for continuous variables as appropriate. Two-tailed *p* values of <0.05 were considered statistically significant.

### Data availability

Anonymized data can be shared by request from any qualified investigator.

## Results

### PCA of clinico-radiologic data within the antibody-negative cohort identifies 3 distinct patient subgroups

**Figure 2:**
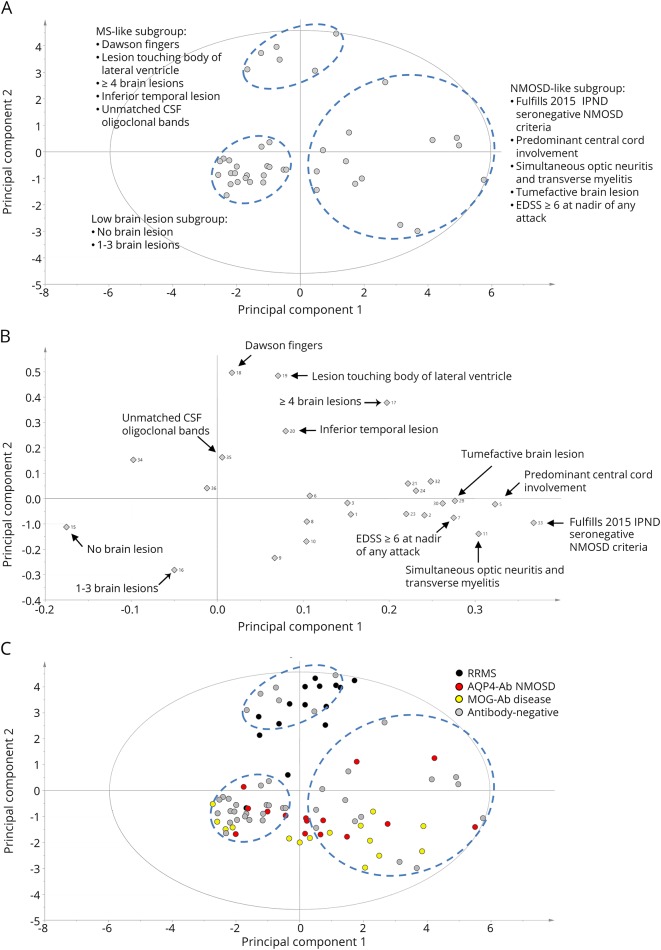
Identification of phenotypic subgroups within the antibody-negative cohort by PCA using clinico-radiologic data (A) Spontaneous separation of antibody-negative patients into 3 distinct clusters using the 36 predefined clinico-radiologic parameters alone (dashed blue circles). (B) Variable loadings plot of the clinico-radiologic parameters allows visualization of parameters responsible for patient clustering. Each parameter is represented by a gray diamond. The number beside each diamond corresponds to the number listed in table e-1 (links.lww.com/NXI/A155). This enables the 3 phenotypic clusters to be classified as an MS-like subgroup, an NMOSD-like subgroup, and an LBL subgroup (panel A inset). (C) Insertion of clinico-radiologic data from the clinical cohort of patients with RRMS, AQP4-Ab NMOSD, and MOG-Ab disease into the PCA scores plot shows corroboration of the phenotypic subgroups with known diagnostic clusters. AQP4-Ab = aquaporin-4 antibody; EDSS = Expanded Disability Status Scale; IPND = International Panel for NMO Diagnosis; LBL = low brain lesion; MOG-Ab = myelin oligodendrocyte glycoprotein antibody; NMOSD = neuromyelitis optica spectrum disorders; PCA = principal component analysis; RRMS = relapsing-remitting MS.

To identify potential phenotypic subgroups within antibody-negative patients, we performed unsupervised PCA of the 36 specified clinico-radiologic parameters and generated a PCA scores plot (figure 2A). Each point in the plot represents all 36 clinico-radiologic parameters from 1 patient; points closer to one another are more clinically alike. Spontaneous separation of the antibody-negative cohort into 3 patient clusters (dashed blue circles) was observed on the PCA plot (figure 2A). This observation suggested a distinct clinical profile for each cluster, and we sought to explore the reason for clustering.

The variable loadings plot of the PCA was constructed to identify the variables driving the clustering (figure 2B). The variables driving the top cluster are features characteristic of MS,^3,24^ whereas the ones defining the bottom right cluster are more typical of NMOSD.^15,16,25^ The bottom left cluster is characterized by no or low brain lesion load. This allowed us to classify these 3 phenotypic clusters into an MS-like subgroup, an NMOSD-like subgroup, and a low brain lesion (LBL) subgroup (figure 2A), with the most principal variables listed in the inset.

To corroborate these phenotypic assignments with patients with established diagnosis, the 36 clinico-radiologic parameters were collected from patients in the clinical cohort of known RRMS and Ab-NMOSD. Insertion of this data set confirmed that most of the patients with RRMS clustered with the MS-like subgroup, whereas the majority of the patients with AQP4-Ab NMOSD and MOG-Ab disease clustered to the NMOSD-like subgroup (figure 2C). It is interesting to note the clustering of patients with AQP4-Ab and MOG-Ab, and this is consistent with previous studies that have shown that AQP4-Ab NMOSD and MOG-Ab disease in adults have largely identical clinical presentations and cannot be distinguished on conventional MRI.^4,26^ Of note, some patients with RRMS, AQP4-Ab NMOSD, and MOG-Ab disease clustered with the LBL subgroup, highlighting that these diseases have overlapping clinico-radiologic features.

Taking these observations in totality, PCA of clinico-radiologic data within the antibody-negative cohort identified 3 phenotypically distinct subgroups: an MS-like subgroup (n = 6), an NMOSD-like subgroup (n = 14), and an LBL subgroup (n = 21). Table 1 shows the demographic and clinical data of the antibody-negative patients grouped by the 3 PCA-defined subgroups and the proportions of patients having each of the 36 clinico-radiologic parameters.

**Table 1:**
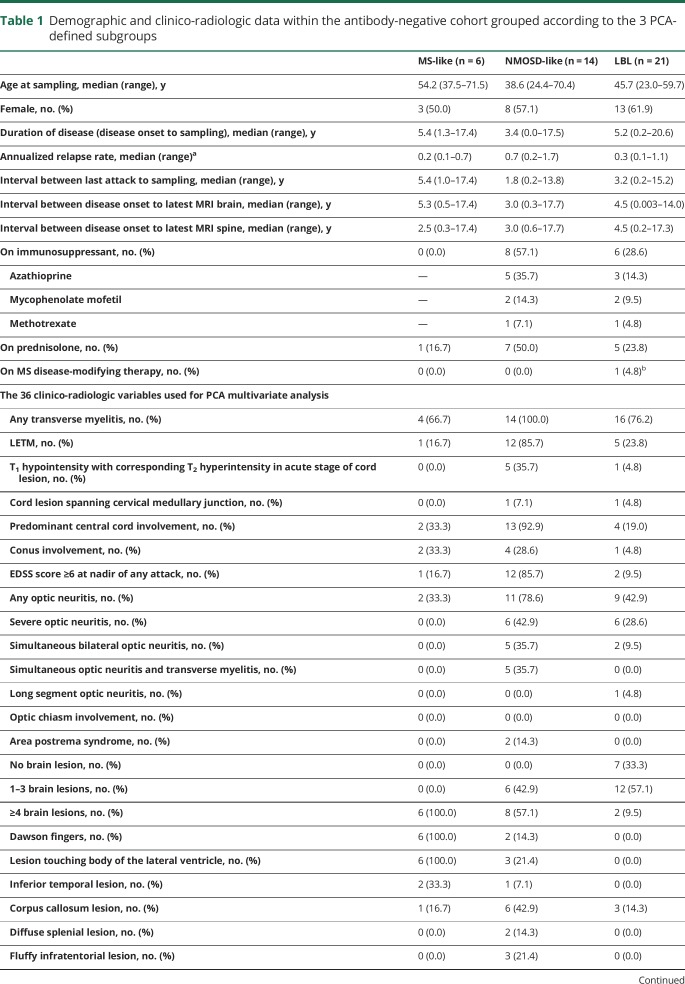

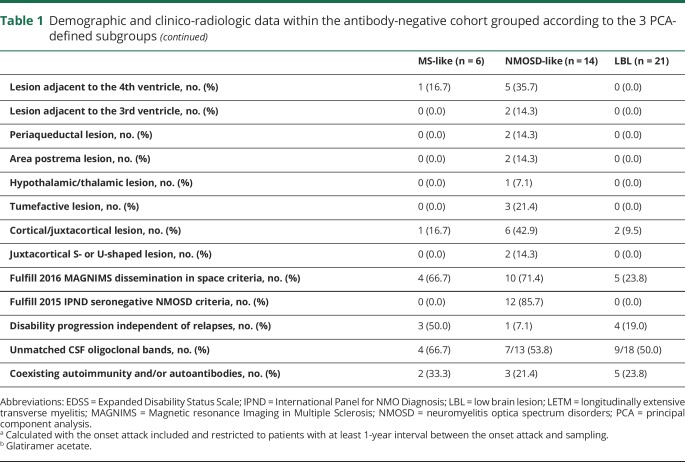
Demographic and clinico-radiologic data within the antibody-negative cohort grouped according to the 3 PCA-defined subgroups

### Plasma myoinositol and formate discriminate between RRMS and Ab-NMOSD with high accuracy within the reference cohort

**Figure 3:**
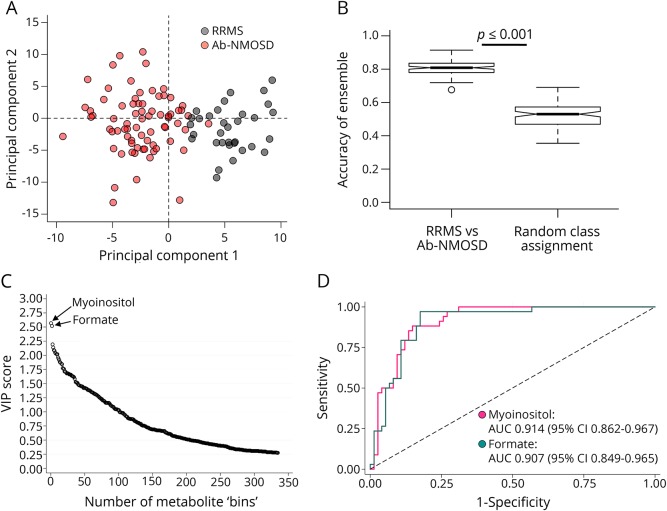
OPLS-DA score plot of metabolomics spectral data comparing RRMS with Ab-NMOSD from the reference cohort (A) OPLS-DA scores plot shows good separation of patients with RRMS from patients with Ab-NMOSD based on metabolomics spectral data. (B) Mean accuracy of the disease groups model is significantly greater than that of the random class assignment model (mean [SD], 80.7% [4.2%] vs 52.3% [7.6%], *p* < 0.001). (C) The top 2 discriminatory metabolites, myoinositol and formate, are identified by their high VIP scores. (D) High AUC of both myoinositol and formate in distinguishing RRMS and Ab-NMOSD. Ab-NMOSD = antibody-positive neuromyelitis optica spectrum disorders; AUC = area under the curve; OPLS-DA = orthogonal partial least square discriminant analysis; RRMS = relapsing-remitting MS; VIP = variable importance in projection.

Although unbiased PCA of extensive clinico-radiologic data is able to identify distinct phenotypes within the antibody-negative cohort, pathophysiologic relevance at a molecular level with respect to the reference diseases (i.e., MS pathology vs antibody-mediated pathology) is lacking. Thus, to investigate whether plasma metabolomics can identify metabolic biomarkers separating the antibody-negative phenotypic subgroups with inference to their underlying pathologies, we obtained discriminatory metabolic markers in the reference cohort of patients with known RRMS and Ab-NMOSD. First, OPLS-DA was used to build discriminatory models using metabolomics spectral data to distinguish between RRMS and Ab-NMOSD within the reference cohort. A representative OPLS-DA scores plot was generated (figure 3A). Each point in the plot represents all metabolomics data from 1 patient; points closer to one another are more metabolically similar. A clear separation between RRMS and Ab-NMOSD was observed on the scores plot. This separation was validated as the mean accuracy (of the ensemble of accuracies) of the disease groups model was significantly greater than the mean accuracy of the random class assignment model (mean [SD], 80.7% [4.2%] vs 52.3% [7.6%]; *p* < 0.001) (figure 3B). No potential confounders were identified within this data set after extensive investigation as reported previously.^2^

Next, to identify the most important metabolites driving the separation between RRMS and Ab-NMOSD, variable importance in projection (VIP) scores were generated. A VIP score is a measure of a variable's importance to the OPLS-DA model; the higher the VIP score, the greater the contribution a variable makes to the model. Ranking of VIP scores revealed that myoinositol and formate (both metabolites being higher in RRMS) were the 2 most important metabolites driving this separation (figure 3C), with a VIP score of 2.57 and 2.51, respectively. Receiver operating characteristic analysis revealed high diagnostic accuracies, as measured by the area under the curve (AUC) of myoinositol (AUC 0.914, 95% CI 0.862–0.967) and formate (AUC 0.907, 95% CI 0.849–0.965) (figure 3D).

### Myoinositol and formate levels are significantly higher in the MS-like subgroup compared with the NMOSD-like subgroup within the antibody-negative cohort

**Figure 4:**
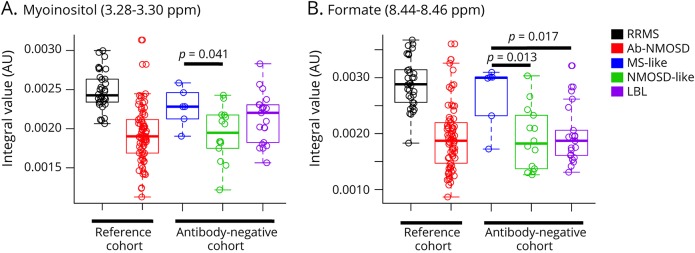
Boxplots comparing myoinositol and formate levels between MS-like and NMOSD-like subgroups within the antibody-negative cohort Both (A) myoinositol and (B) formate are significantly higher in the MS-like subgroup compared with the NMOSD-like subgroup. On one-way ANOVA, (B) formate was significantly different across the 3 subgroups, and post hoc comparisons using the Tukey HSD test showed formate to be significantly higher in the MS-like subgroup compared with the NMOSD-like subgroup, as well as to the LBL subgroup. *p* values shown in (B) are from one-way ANOVA with post hoc multiple comparison corrections. Boxplots of myoinositol and formate in patients with RRMS and Ab-NMOSD are constructed from the same data used to generate the AUC graphs in figure 3D. Ab-NMOSD = antibody-positive NMOSD; ANOVA = analysis of variance; AU = arbitrary units; AUC = area under the curve; LBL = low brain lesion; NMOSD = neuromyelitis optica spectrum disorders; ppm = parts per million; RRMS = relapsing-remitting MS.

**Figure 5:**
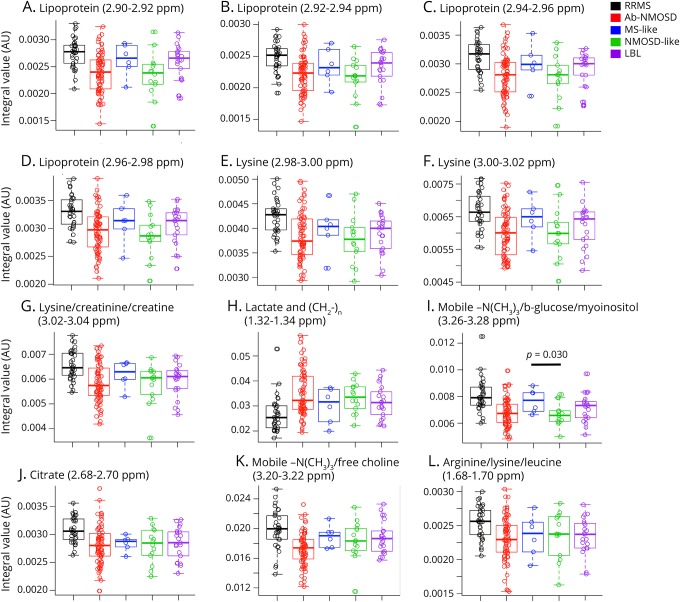
Boxplots of other discriminatory metabolites (VIP score ≥1.75) Other discriminatory metabolites trend in the same direction when comparing the MS-like with NMOSD-like subgroups, as with RRMS to Ab-NMOSD (A-I). This trend becomes less clear with lower VIP scores as shown by the last 3 metabolite bins in the panel; (J) citrate (2.68–2.70 ppm, VIP score 1.87), (K) mobile–N(CH_3_)_3_/free choline (3.20–3.22 ppm, VIP score 1.85), and (L) arginine/lysine/leucine (1.68–1.70 ppm, VIP score 1.75). Ab-NMOSD = antibody-positive NMOSD; AU = arbitrary units; LBL = low brain lesion; NMOSD = neuromyelitis optica spectrum disorders; ppm = parts per million; RRMS = relapsing-remitting MS; VIP = variable importance in projection.

As myoinositol and formate could accurately discriminate between RRMS and Ab-NMOSD, we explored whether these metabolites are different between the MS-like and NMOSD-like clinico-radiologic subgroups within the antibody-negative cohort. Myoinositol was significantly higher in the MS-like subgroup compared with the NMOSD-like subgroup (mean [SD], 0.0023 [0.0002] vs 0.0019 [0.0003] AU; *p* = 0.041) (figure 4A). Formate was also significantly elevated in the MS-like subgroup vs the NMOSD-like subgroup (0.0027 [0.0006] vs 0.0019 [0.0006] AU; *p* = 0.010). On one-way ANOVA, formate was significantly different across the 3 subgroups [F(2,38) = 5.02; *p* = 0.012]; post hoc comparisons using the Tukey HSD test showed formate to be higher in the MS-like subgroup compared with the NMOSD-like subgroup (*p* = 0.013), as indeed compared with the LBL subgroup (0.0027 [0.0006] vs 0.0020 [0.0005] AU; *p* = 0.017) (figure 4B). Taking successive discriminatory metabolites with cutoff VIP scores ≥1.75 (before the second drop-off in VIP scores, see figure 3C) showed similar trends in separating the MS-like from NMOSD-like subgroups (figure 5). Next, we explored whether the MS-like and NMOSD-like patients were metabolically similar to patients with RRMS and Ab-NMOSD, respectively. Using metabolomics spectral data, we were unable to distinguish MS-like patients from patients with RRMS and NMOSD-like patients from patients with Ab-NMOSD (figure e-1, links.lww.com/NXI/A154).

In summary, the 2 most discriminatory metabolites obtained from the OPLS-DA model of RRMS vs Ab-NMOSD were also significantly different between the MS-like and NMOSD-like subgroups (and in the same direction) within antibody-negative patients. This suggests that the MS-like and NMOSD-like subgroups have different underlying pathologies, akin to their respective reference diseases (i.e., RRMS and antibody-mediated NMOSD).

### Differences in myoinositol and formate levels are not accounted for by potential confounders

As a higher proportion of patients in the NMOSD-like and LBL subgroups were on immunosuppressants and prednisolone at the time of plasma sampling compared with the MS-like subgroup (table 1), it was explored whether these accounted for the differences in myoinositol and formate levels. By combining the NMOSD-like and LBL subgroups, myoinositol and formate levels of patients on immunosuppressants were compared with patients not on immunosuppressants. Similar analysis was performed for prednisolone use. There were no statistically significant differences in both metabolites stratified by immunosuppressant or prednisolone use; myoinositol by immunosuppressant use (on immunosuppressant, 0.0020 [0.0002] vs off immunosuppressant, 0.0021 [0.0004] AU; *p* = 0.384); myoinositol by prednisolone use (on prednisolone, 0.0020 [0.0004] vs off prednisolone, 0.0021 [0.0003] AU; *p* = 0.224); formate by immunosuppressant use (on immunosuppressant, 0.0019 [0.0005] vs off immunosuppressant, 0.0020 [0.0005] AU; *p* = 0.714); and formate by prednisolone use (on prednisolone, 0.0017 [0.0005] vs off prednisolone, 0.0020 [0.0005] AU; *p* = 0.111). In fact within the NMOSD-like subgroup alone, patients on immunosuppressants had higher levels of myoinositol (on immunosuppressant, 0.0020 [0.0002] vs off immunosuppressant, 0.0018 [0.0005] AU; *p* = 0.370) and formate (on immunosuppressant, 0.0021 [0.0006] vs off immunosuppressant, 0.0016 [0.0002] AU; *p* = 0.143), and this would, if anything, reduced the discriminatory power of the metabolites. Similar analyses were performed for age, sex, disease duration, and interval since last attack with no significant differences/correlations in the levels of both metabolites based on these parameters (data not shown).

## Discussion

Our findings confirmed that distinct phenotypic subgroups exist within the antibody-negative cohort using advanced PCA pattern-recognition techniques coupled with extensive clinico-radiologic data, without a priori assumptions of their clinical diagnosis. We then applied the 2 metabolites that were the most discriminatory between RRMS and Ab-NMOSD and confirmed that these same metabolites distinguished between the antibody-negative subgroups that were MS-like and NMOSD-like. This suggests that the clinico-radiologic separation by PCA is pathophysiologically meaningful, and we suggest that in clinical practice, the features shown in figure 2A (inset) are pathologically relevant for classification. This has the potential to help guide treatment decisions when seeing antibody-negative patients in the clinic.

Myoinositol is a component of the cell membrane and myelin and is involved in intracellular signaling in many CNS cells.^27^ More importantly, it has been recognized as a marker of astrocyte activation and proliferation.^28^ Low myoinositol levels have been observed in AQP4-Ab NMOSD compared with MS after transverse myelitis using ^1^H magnetic resonance spectroscopy (MRS) of the spinal cord, reflecting astrocytic necrosis.^29^ Conversely, high myoinositol levels have been noted in RRMS and clinically isolated syndrome compared with controls using ^1^H MRS of normal-appearing white matter, indicating astrocytosis and astrogliosis.^30,31^ Unlike AQP4-Ab NMOSD, MOG-Ab disease is not an astrocytopathy and glial fibrillary acidic protein is not elevated in the CSF.^32^ Although accurate quantification of astrocytes has not been performed in MOG-Ab disease in view of the small number of cases with histopathology, it is likely that extent of gliosis as seen in MS (resulting from ongoing chronic neuroinflammation) does not occur in MOG-Ab disease,^33^ and this may explain the reduced levels of myoinositol with respect to MS. This needs further pathologic verification. Our findings of higher myoinositol levels in RRMS and MS-like patients compared with Ab-NMOSD and NMOSD-like patients are in agreement with these observations. Formate causes mitochondrial damage by inhibiting cytochrome c oxidase resulting in disruption of the electron transport chain and production of reactive oxygen species.^34^ Formate-induced cytotoxicity has been demonstrated in rat hippocampal cultures and in retinal (human and rat) cell cultures.^35,36^ Of interest, methanol poisoning is mediated by formate, producing optic nerve demyelination and subsequent progressive retinal axonal loss in humans.^37,38^ As mitochondrial dysfunction has been implicated in MS pathogenesis, it is of interest to note the higher formate levels in patients with MS.^39^ How formate is involved in this process, if at all, as a primary mediator or as part of an injurious cascade will require further mechanistic studies.

In view of the lack of accuracy of the McDonald criteria to separate MS from NMOSD,^40–42^ we have previously attempted to better delineate MS from Ab-NMOSD using conventional MRI parameters.^3,4^ Distinctive MRI brain features of MS include Dawson fingers, inferior temporal lobe lesion, and lesion adjacent to the body of the lateral ventricle,^3,4^ which are also the variables driving the MS-like subgroup in this current study. We have previously shown that blood-based metabolomics can accurately separate MS from controls and from AQP4-Ab NMOSD and MOG-Ab disease.^2,43^ The current study combines both approaches by using metabolomics to give pathologic support to the spontaneously separating clinico-radiologic phenotypes. Of note, the clinico-MRI phenotypic classification identified the 2015 seronegative NMOSD criteria as the most important distinguishing NMOSD-like variable, independently supporting these criteria.

Our study is limited by the small sample size due to the rarity of antibody-negative patients; however, we were still able to show a remarkable similar pattern of discriminatory metabolites in the MS-like against the NMOSD-like subgroups, as seen in patients with RRMS against patients with Ab-NMOSD. Our methodology is optimized to compare 2 subsets, and in the antibody-negative group, there will be multiple disorders; hence, we focused on the 2 phenotypic subgroups, which appeared to represent MS-like and antibody-mediated–like pathology. The third phenotypic subgroup in our analysis contained patients with lower brain lesion load without any MS-like or NMOSD-like discriminators, and pathologies among this subgroup will include antibody-mediated pathologies, MS, other cell-mediated disorders such as CNS sarcoidosis, and monophasic postinfectious conditions. In view of the mixed conditions within the LBL subgroup, we have kept it separate for analysis. Clinicopathologic classification within this LBL subgroup will be particularly challenging. However, in patients with 1–3 brain lesions who have MS-like or NMOSD-like discriminators, these clinico-radiologic discriminators are still potentially useful, as illustrated by 43% of NMOSD-like patients having 1–3 brain lesions. Future validation of our findings is needed in an independent cohort of antibody-negative patients.

Our study demonstrates the strength of computational modeling of clinico-MRI features, which cannot be done in a consistent and unbiased way by clinicians in the clinical setting, given the huge amount of data available for each patient. We also demonstrate the use of metabolomics in supporting the results of such analysis. We have selected a diagnostically challenging group of patients and have been able to identify useful clinical and radiologic characteristics that support some individuals having likely MS and others with likely antibody-mediated pathology. As the MRI parameters are not time restricted, these observations are more useful to apply in clinical practice. Prospective work to study treatment responses and long-term outcome, along with CSF metabolomics analysis and samples taken during relapses, may further improve this classification, especially in patients within the LBL subgroup.

## Glossary

Ab-NMOSD: antibody-positive NMOSD
ANOVA: analysis of variance
AQP4-Ab: aquaporin-4 antibody
AU: arbitrary units
AUC: area under the curve
CPMG: Carr-Purcell-Meiboom-Gill
LBL: low brain lesion
MOG-Ab: myelin oligodendrocyte glycoprotein antibody
MRS: magnetic resonance spectroscopy
NMOSD: neuromyelitis optica spectrum disorders
OPLS-DA: orthogonal partial least square discriminant analysis
PCA: principal component analysis
ppm: parts per million
RRMS: relapsing-remitting MS
VIP: variable importance in projection

## References

[1] Jurynczyk M, Weinshenker B, Akman-Demir G, et al Status of diagnostic approaches to AQP4-IgG seronegative NMO and NMO/MS overlap syndromes. J Neurol 2016;263:140–149. 26530512 10.1007/s00415-015-7952-8 PMC4816597

[2] Jurynczyk M, Probert F, Yeo T, et al Metabolomics reveals distinct, antibody-independent, molecular signatures of MS, AQP4-antibody and MOG-antibody disease. Acta Neuropathol Commun 2017;5:95. 29208041 10.1186/s40478-017-0495-8 PMC5718082

[3] Matthews L, Marasco R, Jenkinson M, et al Distinction of seropositive NMO spectrum disorder and MS brain lesion distribution. Neurology 2013;80:1330–1337. 23486868 10.1212/WNL.0b013e3182887957 PMC3656462

[4] Jurynczyk M, Geraldes R, Probert F, et al Distinct brain imaging characteristics of autoantibody-mediated CNS conditions and multiple sclerosis. Brain 2017;140:617–627. 28364548 10.1093/brain/aww350

[5] Palace J, Leite MI, Nairne A, Vincent A Interferon beta treatment in neuromyelitis optica: increase in relapses and aquaporin 4 antibody titers. Arch Neurol 2010;67:1016–1017. 20697055 10.1001/archneurol.2010.188

[6] Kleiter I, Hellwig K, Berthele A, et al Failure of natalizumab to prevent relapses in neuromyelitis optica. Arch Neurol 2012;69:239–245. 22332191 10.1001/archneurol.2011.216

[7] Stellmann JP, Krumbholz M, Friede T, et al Immunotherapies in neuromyelitis optica spectrum disorder: efficacy and predictors of response. J Neurol Neurosurg Psychiatry 2017;88:639–647. 28572277 10.1136/jnnp-2017-315603 PMC5537514

[8] Min JH, Kim BJ, Lee KH Development of extensive brain lesions following fingolimod (FTY720) treatment in a patient with neuromyelitis optica spectrum disorder. Mult Scler 2012;18:113–115. 22146605 10.1177/1352458511431973

[9] Shimizu J, Hatanaka Y, Hasegawa M, et al IFNbeta-1b may severely exacerbate Japanese optic-spinal MS in neuromyelitis optica spectrum. Neurology 2010;75:1423–1427. 20826711 10.1212/WNL.0b013e3181f8832e

[10] Azzopardi L, Cox AL, McCarthy CL, Jones JL, Coles AJ Alemtuzumab use in neuromyelitis optica spectrum disorders: a brief case series. J Neurol 2016;263:25–29. 26477020 10.1007/s00415-015-7925-y

[11] Wildemann B, Jarius S, Schwarz A, et al Failure of alemtuzumab therapy to control MOG encephalomyelitis. Neurology 2017;89:207–209. 28600462 10.1212/WNL.0000000000004087

[12] Yamout BI, Beaini S, Zeineddine MM, Akkawi N Catastrophic relapses following initiation of dimethyl fumarate in two patients with neuromyelitis optica spectrum disorder. Mult Scler 2017;23:1297–1300. 28391740 10.1177/1352458517694086

[13] Waters P, Woodhall M, O'Connor KC, et al MOG cell-based assay detects non-MS patients with inflammatory neurologic disease. Neurol Neuroimmunol Neuroinflamm 2015;2:e89 doi: 10.1212/NXI.0000000000000089. 25821844 10.1212/NXI.0000000000000089 PMC4370386

[14] Waters PJ, McKeon A, Leite MI, et al Serologic diagnosis of NMO: a multicenter comparison of aquaporin-4-IgG assays. Neurology 2012;78:665–671. 22302543 10.1212/WNL.0b013e318248dec1 PMC3286228

[15] Jurynczyk M, Craner M, Palace J Overlapping CNS inflammatory diseases: differentiating features of NMO and MS. J Neurol Neurosurg Psychiatry 2015;86:20–25. 25248365 10.1136/jnnp-2014-308984

[16] Kim HJ, Paul F, Lana-Peixoto MA, et al MRI characteristics of neuromyelitis optica spectrum disorder: an international update. Neurology 2015;84:1165–1173. 25695963 10.1212/WNL.0000000000001367 PMC4371410

[17] Lenz EM, Bright J, Wilson ID, Morgan SR, Nash AF A 1H NMR-based metabonomic study of urine and plasma samples obtained from healthy human subjects. J Pharm Biomed Anal 2003;33:1103–1115. 14656601 10.1016/s0731-7085(03)00410-2

[18] Tang H, Wang Y, Nicholson JK, Lindon JC Use of relaxation-edited one-dimensional and two dimensional nuclear magnetic resonance spectroscopy to improve detection of small metabolites in blood plasma. Anal Biochem 2004;325:260–272. 14751261 10.1016/j.ab.2003.10.033

[19] Wishart DS, Jewison T, Guo AC, et al HMDB 3.0—the human metabolome database in 2013. Nucleic Acids Res 2013;41:D801–D807. 23161693 10.1093/nar/gks1065 PMC3531200

[20] Wishart DS, Knox C, Guo AC, et al HMDB: a knowledgebase for the human metabolome. Nucleic Acids Res 2009;37:D603–D610. 18953024 10.1093/nar/gkn810 PMC2686599

[21] Wishart DS, Tzur D, Knox C, et al HMDB: the human metabolome database. Nucleic Acids Res 2007;35:D521–D526. 17202168 10.1093/nar/gkl923 PMC1899095

[22] Worley B, Powers R PCA as a practical indicator of OPLS-DA model reliability. Curr Metabolomics 2016;4:97–103. 27547730 10.2174/2213235X04666160613122429 PMC4990351

[23] Thevenot EA, Roux A, Xu Y, Ezan E, Junot C Analysis of the human adult urinary metabolome variations with age, body mass index, and gender by implementing a comprehensive workflow for univariate and OPLS statistical analyses. J Proteome Res 2015;14:3322–3335. 26088811 10.1021/acs.jproteome.5b00354

[24] Arrambide G, Tintore M, Espejo C, et al The value of oligoclonal bands in the multiple sclerosis diagnostic criteria. Brain 2018;141:1075–1084. 29462277 10.1093/brain/awy006

[25] Wingerchuk DM, Banwell B, Bennett JL, et al International consensus diagnostic criteria for neuromyelitis optica spectrum disorders. Neurology 2015;85:177–189. 26092914 10.1212/WNL.0000000000001729 PMC4515040

[26] Hyun JW, Woodhall MR, Kim SH, et al Longitudinal analysis of myelin oligodendrocyte glycoprotein antibodies in CNS inflammatory diseases. J Neurol Neurosurg Psychiatry 2017;88:811–817. 28684532 10.1136/jnnp-2017-315998

[27] Rae CD A guide to the metabolic pathways and function of metabolites observed in human brain 1H magnetic resonance spectra. Neurochem Res 2014;39:1–36. 24258018 10.1007/s11064-013-1199-5

[28] Harris JL, Choi IY, Brooks WM Probing astrocyte metabolism in vivo: proton magnetic resonance spectroscopy in the injured and aging brain. Front Aging Neurosci 2015;7:202. 26578948 10.3389/fnagi.2015.00202 PMC4623195

[29] Ciccarelli O, Thomas DL, De Vita E, et al Low myo-inositol indicating astrocytic damage in a case series of neuromyelitis optica. Ann Neurol 2013;74:301–305. 23553900 10.1002/ana.23909

[30] Chard DT, Griffin CM, McLean MA, et al Brain metabolite changes in cortical grey and normal-appearing white matter in clinically early relapsing-remitting multiple sclerosis. Brain 2002;125:2342–2352. 12244090 10.1093/brain/awf240

[31] Fernando KT, McLean MA, Chard DT, et al Elevated white matter myo-inositol in clinically isolated syndromes suggestive of multiple sclerosis. Brain 2004;127:1361–1369. 15128615 10.1093/brain/awh153

[32] Kaneko K, Sato DK, Nakashima I, et al Myelin injury without astrocytopathy in neuroinflammatory disorders with MOG antibodies. J Neurol Neurosurg Psychiatry 2016;87:1257–1259. 26800711 10.1136/jnnp-2015-312676

[33] Shu Y, Long Y, Wang S, et al Brain histopathological study and prognosis in MOG antibody-associated demyelinating pseudotumor. Ann Clin Transl Neurol 2019;6:392–396. 30847372 10.1002/acn3.712 PMC6389737

[34] Nicholls P The effect of formate on cytochrome aa3 and on electron transport in the intact respiratory chain. Biochim Biophys Acta 1976;430:13–29. 4141 10.1016/0005-2728(76)90218-8

[35] Kapur BM, Vandenbroucke AC, Adamchik Y, Lehotay DC, Carlen PL Formic acid, a novel metabolite of chronic ethanol abuse, causes neurotoxicity, which is prevented by folic acid. Alcohol Clin Exp Res 2007;31:2114–2120. 18034701 10.1111/j.1530-0277.2007.00541.x

[36] Treichel JL, Henry MM, Skumatz CM, Eells JT, Burke JM Formate, the toxic metabolite of methanol, in cultured ocular cells. Neurotoxicology 2003;24:825–834. 14637377 10.1016/S0161-813X(03)00059-7

[37] Sharpe JA, Hostovsky M, Bilbao JM, Rewcastle NB Methanol optic neuropathy: a histopathological study. Neurology 1982;32:1093–1100. 6889696 10.1212/wnl.32.10.1093

[38] Nurieva O, Diblik P, Kuthan P, et al Progressive chronic retinal axonal loss following acute methanol-induced optic neuropathy: four-year prospective cohort study. Am J Ophthalmol 2018;191:100–115. 29709459 10.1016/j.ajo.2018.04.015

[39] Witte ME, Mahad DJ, Lassmann H, van Horssen J Mitochondrial dysfunction contributes to neurodegeneration in multiple sclerosis. Trends Mol Med 2014;20:179–187. 24369898 10.1016/j.molmed.2013.11.007

[40] Pittock SJ, Lennon VA, Krecke K, Wingerchuk DM, Lucchinetti CF, Weinshenker BG Brain abnormalities in neuromyelitis optica. Arch Neurol 2006;63:390–396. 16533966 10.1001/archneur.63.3.390

[41] Chan KH, Tse CT, Chung CP, et al Brain involvement in neuromyelitis optica spectrum disorders. Arch Neurol 2011;68:1432–1439. 22084126 10.1001/archneurol.2011.249

[42] Asgari N, Lillevang ST, Skejoe HP, Falah M, Stenager E, Kyvik KO A population-based study of neuromyelitis optica in Caucasians. Neurology 2011;76:1589–1595. 21536639 10.1212/WNL.0b013e3182190f74 PMC3269768

[43] Dickens AM, Larkin JR, Griffin JL, et al A type 2 biomarker separates relapsing-remitting from secondary progressive multiple sclerosis. Neurology 2014;83:1492–1499. 25253748 10.1212/WNL.0000000000000905 PMC4222850

